# The Influence of Recombinant Human Erythropoietin on Apoptosis and Cytokine Production of CD4^+^ lymphocytes from Hemodialyzed Patients

**DOI:** 10.1007/s10875-012-9835-4

**Published:** 2012-11-20

**Authors:** Katarzyna A. Lisowska, Alicja Dębska-Ślizień, Aleksandra Jasiulewicz, Agnieszka Daca, Ewa Bryl, Jacek M. Witkowski

**Affiliations:** 1 Department of Pathophysiology, Medical University of Gdańsk, Dębinki 7, 80-211 Gdańsk, Poland; 2 Department of Nephrology, Transplantology and Internal Diseases, Medical University of Gdańsk, Gdańsk, Poland

**Keywords:** CD4^+^ lymphocytes, apoptosis, cytokines, rhEPO

## Abstract

Recombinant human erythropoietin (rhEPO) treatment of hemodialyzed (HD) patients normalizes the altered phenotype of CD4^+^ lymphocytes and restores the balance of Th1/Th2 cytokines. We decided to test how the presence of rhEPO in cell culture modulates cytokine production of CD4^+^ lymphocytes in HD patients with stable hemoglobin level and expression of activation antigens of stimulated CD4^+^ lymphocytes similar to those observed in healthy individuals. We also tested whether the presence of rhEPO in cell culture protects stimulated CD4^+^ lymphocytes of HD patients from apoptosis. Peripheral blood mononuclear cells (PBMC) of HD patients were stimulated with an immobilized anti-CD3 antibody with or without addition of rhEPO. The percentage of apoptotic CD4^+^ lymphocytes and the level of Th1/Th2 cytokines in culture supernatants were measured with flow cytometry. HD patients showed a decrease in the percentage of apoptotic CD4^+^ cells after stimulation with the anti-CD3 antibody combined with rhEPO. The level of IFN-γ and IL-10 was increased while the level of TNF-α was decreased in the presence of rhEPO in cell culture from HD patients. These results confirm the role of rhEPO signaling in T lymphocytes of HD patients.

## Introduction

Chronic renal failure is accompanied by a deficiency state in both cell-mediated and humoral immunity which is deepened by hemodialysis (HD) [1]. A direct contact of the patient’s peripheral blood mononuclear cells (PBMC) with an artificial membrane increases the production of pro-inflammatory cytokines: tumor necrosis factor-alpha (TNF-α), interleukin (IL)-1 and IL-6 [2, 3]. What’s more, HD patients demonstrate a decreased production of IL-2 and interferon gamma (IFN-γ) [4, 5]. The deficient production of cytokines is probably related to changes in phenotype of CD4^+^ lymphocytes from HD patients, which exhibit decreased expression of the major co-stimulatory CD28 antigen and main activation markers: CD25 and CD69 [6].

Recombinant human erythropoietin (rhEPO) administered to HD patients to correct the anemia state can influence both the phenotype of T lymphocytes and the production of cytokines. Our previous studies have shown that rhEPO treatment normalizes the impaired expression of CD28 and CD69 antigens of CD4^+^ lymphocytes [7]. Additionally, several months of therapy restores the balance of cytokines by reducing the level of TNF-α [8, 9] and increasing the level of IL-2 and IL-10 [4, 8, 9] in whole blood cell cultures of HD patients during treatment. However, the levels of cytokines change in a different ways during rhEPO treatment. The level of TNF-α and IL-10 changes immediately when the hemoglobin level exceeds the optimal value of 10 g/dl and is stable during treatment, while the level of IL-2 increases continuously during treatment [9]. This observation suggests that the normalization of the levels of TNF-α and IL10 was reached shortly after the start of rhEPO therapy. Increase of the level of IL-2 has been achieved after several months of rhEPO administration, so this change was secondary with respect to changes in the level of TNF-α and IL10. Probably changes in the level of these cytokines are dependent upon various factors. For example, the level of IL-2 may depend on increased expression of CD28 on CD4^+^ lymphocytes and/or decreased percentage of CD152^+^ lymphocytes. However, it is still not clear which cytokines are regulated by the presence of erythropoietin in the circulation.

In order to better understand these mechanisms, we decided to test how the presence of rhEPO in cell culture influences cytokine production in stimulated CD4^+^ lymphocytes from HD patients with a stable hemoglobin level and expression of CD28, CD69 and CD25 antigens of stimulated CD4^+^ lymphocytes similar to those observed in healthy individuals. Repetitive HD procedure has also been reported to induce apoptosis of T lymphocytes, which leads to T-cell lymphopenia sometimes observed in these patients [10]. Therefore, we also tested if the presence of rhEPO in cell culture can protect stimulated CD4^+^ lymphocytes of HD patients from apoptosis.

## Patients and Methods

### Patients

For this study we have chosen 13 HD patients (11 men and 2 women, mean age 59 ± 13.99 years) with a hemoglobin level above 10 g/dl (mean hemoglobin 12.53 ± 1.57 g/dl) and expression of CD28, CD69 and CD25 antigens of stimulated CD4^+^ lymphocytes similar to those observed in healthy controls. 7 HD patients received epoetin alpha or beta and the epoetin doses were adjusted to hemoglobin levels. 6 HD patients who didn’t receive rhEPO were also included in the study group. These patients did not require rhEPO administration, since the level of hemoglobin was maintained at the correct level. They showed similar phenotype of stimulated CD4^+^ lymphocytes and the level of Th1 and Th2 cytokines in culture supernatants as HD patients treated with rhEPO and healthy controls (data not shown). None of the patients suffered from any infection, inflammation, malnutrition, malignancy or blood loss during the study. The study has been approved by the Ethical Committee of the Medical University of Gdańsk.

### Assessment of the Percentage of Apoptotic CD4^+^ lymphocytes and the Level of Th1/Th2 cytokines

Thirty milliliters of venous peripheral blood from each HD patient was collected in tubes containing EDTA as the anti-coagulant agent before HD session. Peripheral blood mononuclear cells (PBMC) were isolated from venous peripheral blood as previously described [6] and stimulated with an immobilized anti-CD3 antibody (125 ng/ml) with or without the addition of rhEPO (epoetin alpha, 0.1 U/ml) and incubated for 3 days at 37 °C, 5 % CO_2_. Stimulated cells were then collected in order to estimate the percentage of apoptotic CD4^+^ lymphocytes. Culture supernatants were collected and frozen at −80 °C for the assessment of cytokine production. Collected cells were stained with the RPE-Cy5-conjugated anti-CD4 monoclonal antibody (DAKO, Denmark) and PE-conjugated annexin V (BD Pharmingen, USA) and analyzed with flow cytometry on FACScan (Becton Dickinson, USA).

Cytometric Bead Array (CBA™, BD Biosciences, USA) was used to estimate the level of Th1/Th2 cytokines produced by stimulated PBMC. Cytokine concentrations were analyzed with the use of Becton Dickinson CBA software.

### Analysis and Statistics

Data were analyzed with Cyflogic, version 1.2.1 (©Perttu Terho and ©CyFlow Ltd). Statistical analysis was done using the Statistica program, version 8 (StatSoft, Poland). The significance tests were chosen according to data distribution. The level of significance in all was *p* ≤ 0.05.

## Results and Discussion

**Fig. 1:**
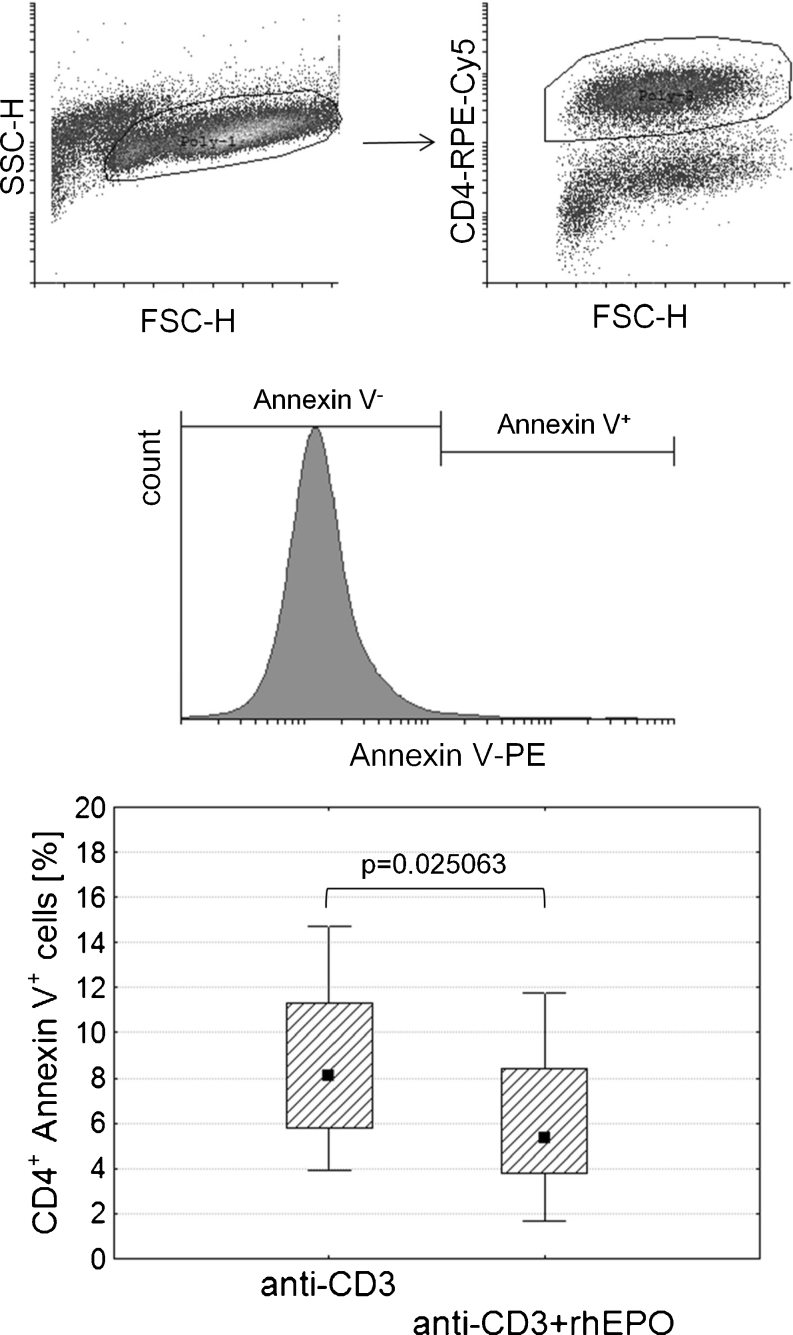
Comparison of the percentage of CD4^+^ cell apoptosis depending on the presence of rhEPO in cell culture. Lymphocytes were selected on the basis of their forward and side scatter characteristics. Annexin V binding assay was used to distinguish between viable and apoptotic cells—apoptotic CD4^+^ lymphocytes were annexin V positive (CD4^+^Annexin V^+^ cells). *Graph* shows the percentage of CD4^+^Annexin V^+^ cells in HD patients. Midpoints of figures present medians, *boxes* present the 25 and 75 percentile and whiskers outside visualize the minimum and maximum of all the data, *p* < 0.05, Wilcoxon signed ranks test

The expression of erythropoietin receptor (EPO-R) has been reported in many non-hematopoietic cells including lymphocytes [11]. We have recently shown that rhEPO increases the phosphorylation of signal transducer and activator of transcription 5 (STAT5) in stimulated CD4^+^ lymphocytes [12]. Phosphorylation of STAT5 in T lymphocytes plays an important role in promoting their survival. Meanwhile, repetitive HD procedure has been reported to induce apoptosis of T lymphocytes [10]. Therefore, we examined whether the presence of rhEPO in cell culture can protect stimulated CD4^+^ lymphocytes of HD patients from apoptosis and we have shown that if present at a concentration similar to the physiological level rhEPO promotes the survival of CD4^+^ lymphocytes. HD patients showed a significant decrease in the percentage of apoptotic CD4^+^ cells (CD4^+^Annexin V^+^ cells) after stimulation with the anti-CD3 antibody combined with rhEPO as compared to cells stimulated with the anti-CD3 antibody alone (Fig. 1). This fact has been observed in other cell types and our team is being the first to described it in T lymphocytes.

We also investigated the level of Th1 cytokines (IL-2, IFN-γ, TNF-α) and Th2 cytokines (IL-4, IL-5, IL-10) in culture supernatants of lymphocytes stimulated with the anti-CD3 antibody with or without addition of rhEPO. We didn’t observe any changes in the level of IL-2 in culture supernatants of lymphocytes stimulated with the anti-CD3 antibody in the presence of rhEPO. Since the phenotype of stimulated CD4^+^ lymphocytes from these patients and the level of IL-2 in culture supernatants is similar to those observed in healthy individuals, we suspect that the production of IL-2 is dependent on the level of CD28 antigen on CD4^+^ lymphocytes, as confirmed by a positive correlation between these parameters (data not shown). This relationship has already been described by other authors [13].

**Fig. 2:**
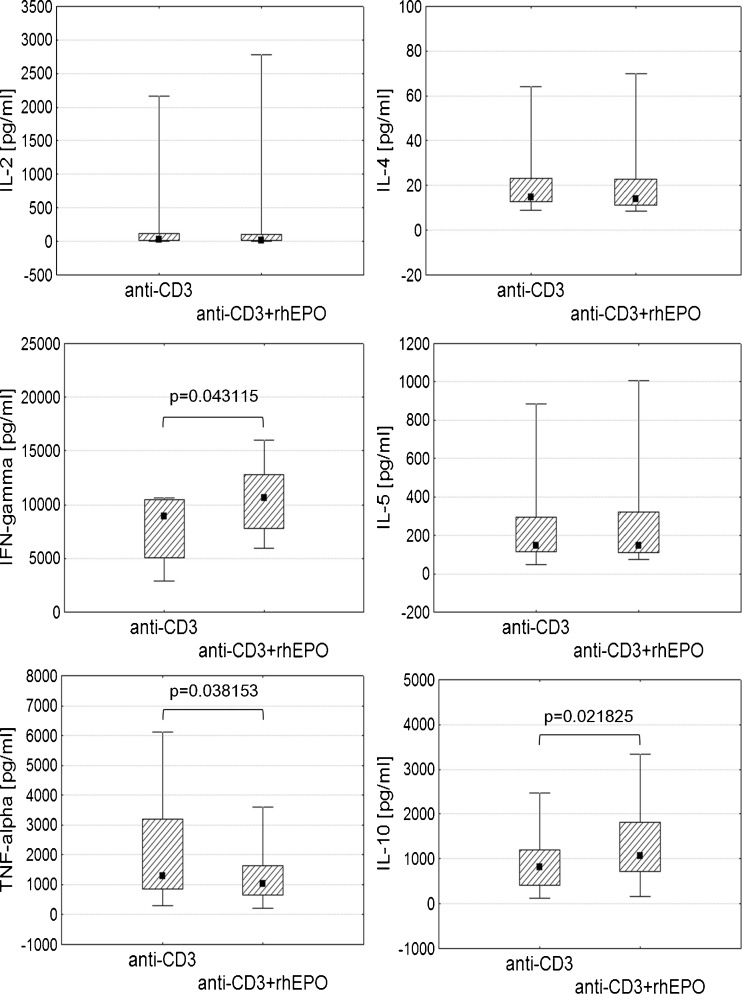
Comparison of the levels of Th1 and Th2 cytokines in culture supernatants of stimulated lymphocytes depending on the presence of rhEPO. *Graphs* show the level of Th1 (IL-2, IFN-γ, TNF-α) and Th2 (IL-4, IL-5, IL-10) cytokines in HD patients. Midpoints of figures present medians, *boxes* present the 25 and 75 percentile and whiskers outside visualize the minimum and maximum of all the data, *p* < 0.05, Wilcoxon signed ranks test

In our study the presence of rhEPO in cell culture increased the levels of IFN-γ and IL-10 (Fig. 2), whose production can also be enhanced by IL-2 [14, 15]. Since IL-2 and EPO act through receptors that belong to the same family and have common signaling pathways [16], we believe that rhEPO acts like IL-2. The level of TNF-α was decreased in the presence of rhEPO in cell culture from HD patients (Fig. 2). It is not clear whether down-regulation of TNF-α was caused by rhEPO or rather IL-10, whose level was increased in the presence of rhEPO. The level of IL-4 and IL-5 remained unchanged (Fig. 2). Not the first time we demonstrate the impact of rhEPO on cytokine levels in HD patients. Our study shows that rhEPO affects the production of cytokines which are found to be regulated through signaling pathways involving STAT5, for example. The presence of rhEPO appears to regulate levels of TNFTNF-α and IL-10, depending on the initial level of cytokines. Similar experiment was carried out in a group of healthy people. Healthy controls had a lower percentage of apoptotic CD4^+^ cells after stimulation with the anti-CD3 antibody compared to HD patients so the presence of rhEPO in cell culture didn’t influence it. Healthy controls also didn’t show any changes in Th1 or Th2 cytokine levels when rhEPO was added to cell culture but at the same time the level of TNF-α was already decreased while the level of IL-10 and IFN-γ was increased compared to HD patients (data not shown).

## Conclusions

These results confirm one more time the role of rhEPO signaling in T lymphocytes of HD patients. In our opinion, rhEPO protects CD4^+^ lymphocytes from apoptosis and restores the balance of cytokines by reducing the level of TNF-α and increasing the level of anti-inflammatory IL-10, though the mechanism of action of rhEPO on T lymphocytes is still unclear. At the same time these results suggest that improved production of IL-2 is not directly dependent on rhEPO presence but seems to be a consequence of a long-term rhEPO treatment. These observations confirm that rhEPO administration not only has a beneficial effect on the red blood cells but also regulates the functioning of immune cells.
